# Renin–angiotensin system inhibitors and immunotherapy outcomes in lung cancer: a systematic review and meta-analysis with complementary transcriptomic analyses

**DOI:** 10.3389/fimmu.2026.1925741

**Published:** 2026-08-19

**Authors:** Qian Li, Xianjun Min, Jing Bai, Xinbo Liu, Chao Ye

**Affiliations:** 1 Department of Thoracic Surgery, Beijing Genertec Aerospace Hospital, Beijing, China; 2 Department of Thoracic Surgery, The Fourth Hospital of Hebei Medical University, Shijiazhuang, Hebei, China; 3 Department of Pharmacy, The Fourth Hospital of Hebei Medical University, Shijiazhuang, Hebei, China; 4 School of Pharmacy, Hebei Medical University, Shijiazhuang, Hebei, China

**Keywords:** cancer-associated fibroblasts, immune checkpoint inhibitors, lung cancer, meta−analysis, renin−angiotensin system inhibitors, tumor microenvironment

## Abstract

**Background:**

The tumor microenvironment has emerged as an important determinant of response to immune checkpoint inhibitors (ICIs), with increasing attention directed toward stromal components such as cancer-associated fibroblasts (CAFs). Experimental evidence suggests that renin–angiotensin system (RAS) signaling may participate in stromal remodeling and immune regulation, raising interest in whether RAS inhibitors could influence immunotherapy outcomes. However, clinical findings remain inconsistent and the extent to which reported associations reflect biological effects rather than study-level bias remains uncertain. This study aimed to systematically evaluate the association between concomitant RAS inhibitor use and survival outcomes in lung cancer patients receiving ICIs while interpreting the findings within a tumor microenvironment-oriented conceptual framework.

**Methods:**

PubMed and Embase were searched from database inception through February 2026. Eligible studies included lung cancer patients receiving ICIs and reported hazard ratios (HRs) for overall survival (OS) and/or progression-free survival (PFS) according to concomitant RAS inhibitor exposure. Random-effects meta-analyses were performed to pool effect estimates. Publication bias and potential small-study effects were evaluated using Egger’s regression and explored using Precision-Effect Test and Precision-Effect Estimate with Standard Error (PET-PEESE).

**Results:**

Thirteen eligible publications involving 46,618 patients were included. Conventional random-effects analyses suggested improved OS (HR 0.74, 95% CI 0.64–0.86) and PFS (HR 0.81, 95% CI 0.68–0.96) among RAS inhibitor users. However, substantial funnel plot asymmetry and significant Egger’s test results for OS indicated possible small-study effects. Exploratory PET-PEESE analyses attenuated the observed associations toward the null (adjusted OS HR 0.99, 95% CI 0.96–1.02; adjusted PFS HR 1.04, 95% CI 0.85–1.27). Subgroup analyses suggested possible heterogeneity across histological and regional categories, although these findings should be interpreted cautiously.

**Conclusions:**

Current evidence does not indicate a consistent survival advantage associated with concomitant RAS inhibitor use in unselected lung cancer populations treated with ICIs. The discrepancy between conventional pooling and exploratory bias-adjusted analyses suggests that the observed survival advantage may be partially attributable to small-study effects and residual confounding rather than a reproducible treatment-enhancing effect. Rather than supporting routine clinical use of RAS inhibitors to enhance immunotherapy efficacy, these findings support further biomarker-informed investigation into stromal and tumor microenvironment contexts that may contribute to differential treatment responses.

**Systematic review registration:**

https://www.crd.york.ac.uk/PROSPERO/, identifier CRD420261369330.

## Introduction

Immune checkpoint inhibitors (ICIs) targeting the programmed cell death protein 1 (PD-1)/programmed cell death-ligand 1 (PD-L1) pathway have transformed the therapeutic landscape of lung cancer by restoring antitumor T-cell activity (1). Nevertheless, durable clinical responses remain limited to a subset of patients, highlighting the need to identify factors that influence therapeutic efficacy beyond tumor-intrinsic characteristics (2). Although biomarkers such as PD-L1 expression and genomic alterations contribute to treatment variability, increasing evidence indicates that the tumor microenvironment (TME) also plays a critical role in determining responsiveness to immunotherapy (2).

Among stromal components within the TME, cancer-associated fibroblasts (CAFs) have emerged as dynamic regulators of immune contexture and therapeutic responsiveness (3–5). Through extracellular matrix remodeling, regulation of tissue architecture, secretion of soluble mediators, and interactions with immune cells, CAFs can influence immune-cell trafficking and contribute to an immune-restrictive microenvironment (3, 5–7). These collective processes are commonly referred to as CAF-mediated remodeling of the TME, which has been implicated in immune exclusion and resistance to anticancer therapy. At the same time, accumulating evidence indicates that CAFs comprise a heterogeneous and context-dependent cell population rather than a uniformly pro-tumorigenic compartment (8).

Interest has therefore expanded toward identifying clinically available agents capable of modifying stromal biology. Renin–angiotensin system (RAS) inhibitors are widely prescribed for the management of hypertension, heart failure, chronic kidney disease, and other cardiovascular disorders because of their well-established cardiovascular and renal protective effects (9). Beyond these conventional indications, increasing evidence has implicated the RAS in tissue remodeling, fibrosis, and multiple aspects of cancer biology (10–13). A recent review further summarized accumulating evidence that pharmacological inhibition of the RAS—including angiotensin-converting enzyme (ACE) inhibitors, angiotensin receptor blockers (ARB), and direct renin inhibitors—may influence tumor development and progression through mechanisms involving fibrosis, angiogenesis, inflammation, and immune regulation (14). Experimental studies have further suggested that angiotensin signaling contributes to extracellular matrix deposition, fibroblast activation, and broader stromal remodeling within tumors (11, 12, 15). Conversely, ACE inhibitors and ARB have been associated with partial normalization of stromal architecture and improved immune accessibility in preclinical models (16, 17).

These observations have generated growing interest in whether concomitant RAS inhibitor use may influence the efficacy of immune checkpoint blockade. Several retrospective clinical studies have reported improved survival outcomes among patients receiving RAS inhibitors during immunotherapy, whereas others have observed neutral associations (18). Interpretation of these findings remains challenging because observational studies are inherently susceptible to confounding, treatment-selection bias, and reporting bias. Moreover, although both non-small cell lung cancer (NSCLC) and small cell lung cancer (SCLC) have become established indications for ICIs in selected clinical settings (1), it remains uncertain whether the association between RAS inhibition and immunotherapy outcomes differs across histological subtypes.

Given these uncertainties, a systematic synthesis of the available evidence is warranted. Therefore, we conducted a systematic review and meta-analysis to evaluate the association between concomitant RAS inhibitor use and survival outcomes in lung cancer patients receiving ICIs. Beyond quantitatively synthesizing the available clinical evidence, we incorporated exploratory assessment of small-study effects and interpreted the findings within a TME-oriented framework that considers—but does not directly examine—the potential contribution of CAF-mediated stromal remodeling.

## Methods

### Study guideline and protocol

This meta-analysis was conducted in accordance with the Preferred Reporting Items for Systematic Reviews and Meta-Analyses (PRISMA) 2020 statement (19). The study protocol was prospectively registered in the International Prospective Register of Systematic Reviews (PROSPERO; registration number: CRD420261369330).

### Literature search

PubMed and Embase were systematically searched from database inception to February 15, 2026. No language restrictions were imposed. Search terms combined controlled vocabulary and free-text terms related to three domains: renin–angiotensin system inhibitors, immune checkpoint inhibitors, and lung cancer. Terms included ACE inhibitors, angiotensin receptor blockers, PD-1/PD-L1 inhibitors, and major lung cancer descriptors. The full search strategy is presented in **Supplementary Table 1**.

To improve completeness, reference lists of eligible publications and related reviews were additionally screened.

### Eligibility criteria

Studies were considered eligible when they fulfilled all of the following conditions:

1. included adult patients with lung cancer receiving ICI-based therapy;
2. evaluated exposure to concomitant RAS inhibition, including ACE inhibitors and/or ARB;
3. included a comparison group without RAS inhibitor exposure;
4. reported overall survival (OS) and/or progression-free survival (PFS) using hazard ratios (HRs) with corresponding 95% confidence intervals (CIs);
5. adopted observational cohort designs or *post hoc* analyses of randomized studies.

Studies were excluded if they were reviews, editorials, case reports, preclinical studies, conference abstracts lacking sufficient data, duplicate cohorts, or did not provide extractable lung cancer–specific estimates.

In this study, RAS inhibitor exposure was defined as treatment with ACE inhibitors and/or ARB. Other agents targeting the broader renin–angiotensin–aldosterone system, including angiotensin receptor–neprilysin inhibitors, mineralocorticoid receptor antagonists, and direct renin inhibitors, were not considered eligible exposures.

### Data extraction and quality assessment

Two investigators independently extracted data using a predefined extraction template. Information collected included study characteristics, demographic and clinical features, histological composition, treatment details, exposure definitions, and adjusted survival estimates.

When multiple effect estimates were available, multivariable-adjusted models were preferentially retained. When a study reported separate adjusted HRs for ACE inhibitor and ARB exposure, these exposure-specific estimates were extracted independently because they represented distinct pharmacological exposure groups reported by the original investigators.

Methodological quality was evaluated using the Newcastle–Ottawa Scale (NOS) (20). Disagreements were resolved through discussion and consensus.

### Statistical analysis

HRs were pooled using random-effects meta-analysis to account for expected clinical and methodological heterogeneity across studies. OS and PFS were analyzed independently. Between-study heterogeneity was evaluated using Cochran’s *Q* statistic and quantified with the *I²* metric.

Prespecified subgroup analyses explored whether pooled estimates differed according to histological composition (NSCLC-only *versus* mixed histology) and geographic region. Formal subgroup comparisons were performed using between-group interaction testing.

Sensitivity analyses consisted of leave-one-out procedures to evaluate whether pooled estimates were disproportionately influenced by individual studies.

Potential reporting bias and small-study effects were assessed using visual inspection of funnel plots together with Egger’s regression test when sufficient studies were available. For outcomes demonstrating evidence of asymmetry, PET-PEESE and trim-and-fill analyses were additionally performed as exploratory approaches to examine the potential influence of small-study effects on pooled estimates. Because both approaches have recognized limitations, particularly under conditions of substantial between-study heterogeneity, these analyses were interpreted as complementary rather than confirmatory.

All statistical analyses were performed using R software (version 4.5.1). A two-sided *P* value <0.05 was considered statistically significant.

### Complementary transcriptomic analyses of *AGTR1*-associated CAF biology

Single-cell RNA sequencing (scRNA-seq) data were obtained from the scCancerExplorer database (https://bianlab.cn/scCancerExplorer/home) using the NSCLC dataset GSE210347 (21). *AGTR1* expression across different cell populations was visualized to identify its predominant cellular source within the TME.

The association between *AGTR1* expression and CAF infiltration was evaluated using The Cancer Genome Atlas (TCGA) lung adenocarcinoma (LUAD) and lung squamous cell carcinoma (LUSC) cohorts through the Sangerbox platform (http://vip.sangerbox.com) (22). CAF infiltration was estimated using the EPIC deconvolution algorithm. The relationship between *AGTR1* expression and stromal abundance was further assessed by correlating *AGTR1* expression with StromalScore in the same TCGA cohorts using Sangerbox.

The correlation between *AGTR1* expression and tumor purity was evaluated using the TIMER 2.0 platform (http://timer.cistrome.org/) based on TCGA LUAD and LUSC datasets (23). In addition, the association between *AGTR1* expression and transforming growth factor-β (TGF-β) signaling activity was analyzed using the IMPACT platform (www.brimpact.cn), which integrates transcriptomic data from the TCGA LUAD and LUSC cohorts (24). Correlation analyses were performed using the default statistical methods implemented by each platform, and two-sided P values <0.05 were considered statistically significant. These analyses were performed independently of the clinical studies included in the meta-analysis and were intended to provide complementary biological context rather than mechanistic validation of the clinical findings.

## Results

### Study selection

**Figure 1:**
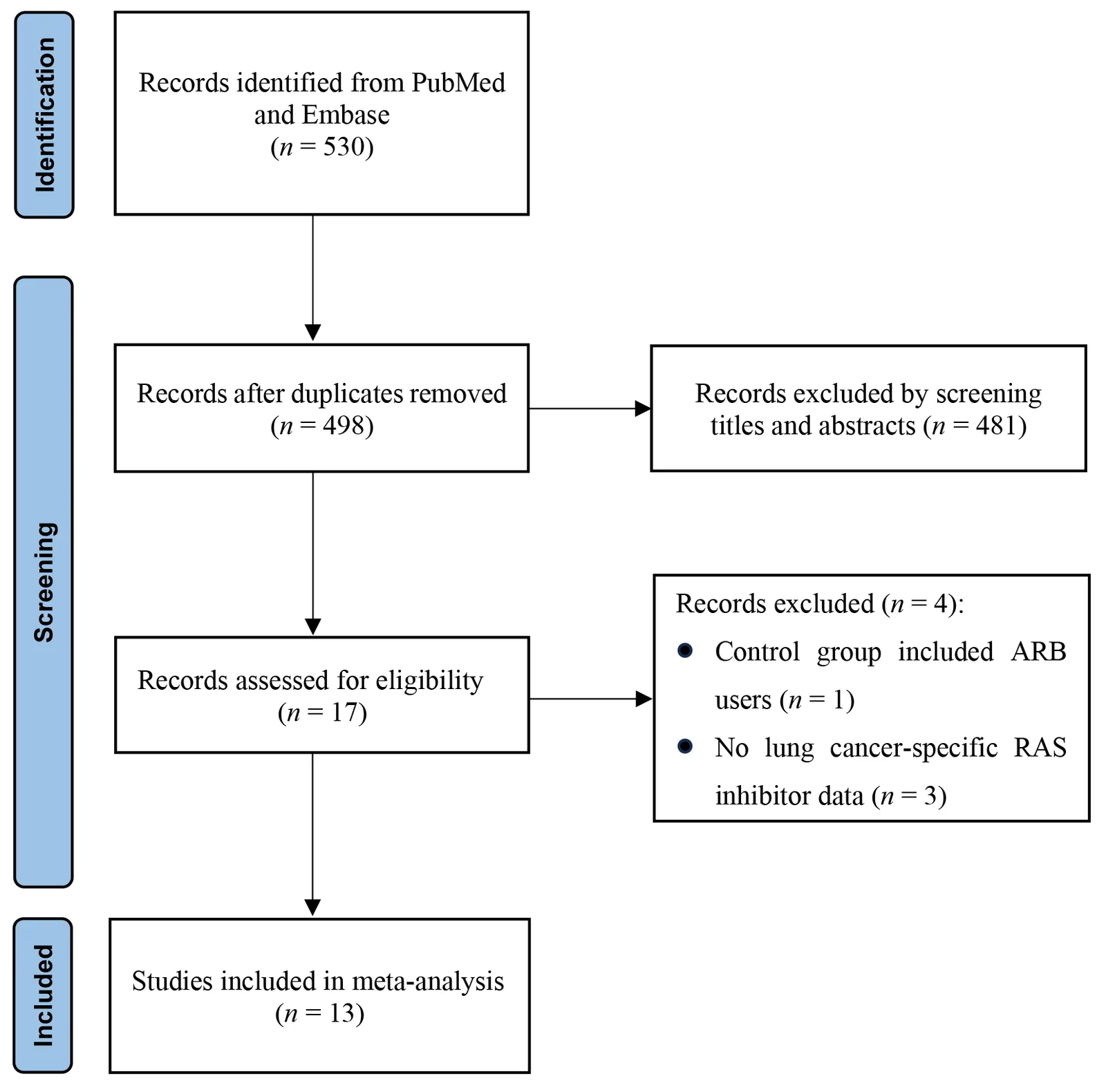
PRISMA flow diagram of study selection.

The literature search yielded 530 records from PubMed and Embase. Following duplicate removal, 498 unique records underwent title and abstract screening. After exclusion of 481 records that did not satisfy the predefined eligibility criteria, 17 studies proceeded to full-text review. Among these, four articles were excluded during eligibility assessment. One study was removed because the comparator arm contained patients exposed to ARB (25), while three studies did not provide extractable outcome data specific to lung cancer populations receiving RAS inhibitors (26–28). Consequently, 13 studies fulfilled all inclusion criteria and were incorporated into the final quantitative synthesis (29–41) The study selection workflow is illustrated in **Figure 1**.

### Study characteristics and quality assessment

The final dataset consisted of 13 eligible studies involving 46,618 patients treated with ICIs for lung cancer. In several included studies, RAS inhibitor exposure was examined as part of broader analyses of concomitant medications rather than as the primary exposure of interest. Detailed characteristics are summarized in **Table 1**.

**Table 1:** Characteristics of studies included in meta-analysis.

Study	Country	Study design	Sample size (user, %)	RASi type and timing	Control definition	Cancer type	ICI regimen	Line of therapy	HR for OS (95% CI)	HR for PFS (95% CI)
Kichenadasse et al., 2021 (29)	Multi-country (trials)	Pooled *post hoc* analysis of 4 RCTs	1548 (358, 23.1)	ACEi or ARB, at trial enrolment	No RASi, other AHs allowed	NSCLC	Atezolizumab (anti-PD-L1)	First or subsequent	0.83 (0.68–1.02)	0.95 (0.83–1.10)
Kostine et al., 2021 (30)	France	Single-center retrospective	150 (NR)	ACEi or ARB, within 1 month before/after ICI start	No RASi, other AHs allowed	NSCLC	Anti-PD-(L)1 Anti-CTLA-4	First or subsequent	0.78 (0.51–1.19)	1.04 (0.69–1.57)
Miura et al., 2021 (31)	Japan	Single-center retrospective	300 (40, 13.3)	ARB only, at ICI initiation	No ARB (ACEi use not reported; may be allowed)	NSCLC	Nivolumab or pembrolizumab	Mostly ≥2nd	0.69 (0.31–1.50)	NR
Pereira et al., 2021 (32)	Portugal	Single-center retrospective	107 (15, 14.0)	ARB, at ICI initiation	No RASi	NSCLC	Anti-PD-(L)1	NR	0.44 (0.19–1.02)	0.40 (0.17–0.93)
			112 (20, 14.0)	ACEi, at ICI initiation					0.75 (0.39–1.32)	0.87 (0.46–1.65)
Tozuka et al., 2021 (33)	Japan	Single-center retrospective	256 (37, 14.5)	ACEi or ARB, at ICI initiation	No RASi, other AHs allowed	NSCLC	Nivolumab, pembrolizumab, or atezolizumab	≥2nd	0.71 (0.45–1.11)	0.65 (0.44–0.98)
Chiang et al., 2023 (34)	China	Two-center retrospective	337 (242, 28.2)	ACEi or ARB, after ICI initiation	No RASi, other AHs allowed	LC	Anti-PD-(L)1	NR	0.36 (0.23–0.57)	0.55 (0.38–0.78)
Zhang et al., 2023 (35)	China	Single−center retrospective	208 (62, 29.8)	ACEi, at ICI initiation or during treatment	No ACEi	SCLC	Durvalumab or atezolizumab (PD-L1 inhibitors)	First or subsequent	0.82 (0.53–1.27)	1.14 (0.82–1.59)
Ma et al., 2024 (36)	China	Two−center retrospective	124 (NR)	ACEi or ARB, at ICI initiation	No RASi, other AHs allowed	LC	Anti-PD-(L)1	First or subsequent	0.39 (0.11–1.40)	0.37 (0.17–0.79)
Rousseau et al., 2024 (37)	France	Nationwide retrospective cohort study	38176 (13882, 36.4%)	ACEi or ARB, at lung cancer diagnosis	No RASi use at diagnosis, other AHs allowed	NSCLC	Pembrolizumab	First	1.01 (0.98–1.04)	NR
Brinzevich et al., 2025 (38)	United States	Retrospective cohort study (VHA national database)	3739 (1101, 29.4%)	ACEi or ARB, within 3 months before ICI start	No RASi, other AHs allowed	NSCLC	Pembrolizumab or nivolumab	First or second line	0.95 (0.88–1.04)	NR
Gobbini et al., 2025 (39)	France	Retrospective cohort study (nationwide cohort)	753 (183, 24.3%)	ACEi or ARB, within 90 days before to 30 days after ICI start	No RASi	NSCLC	Nivolumab	≥2nd	0.78 (0.65–0.93)	0.86 (0.71–1.03)
Efil et al., 2026 (40)	Turkey	Single−center retrospective	286 (94, 32.9%)	ACEi or ARB, at ICI initiation	No RASi, other AHs allowed	LC	Anti-PD-(L)1 Anti-CTLA-4	First or second	0.58 (0.43–0.80)	0.76 (0.57–1.00)
Mutlu Ozkan et al., 2026 (41)	Turkey	Multicenter retrospective	614 (288, 46.9%)	ACEi or ARB, at ICI initiation	No RASi, other AHs allowed	NSCLC	Nivolumab	Second	0.60 (0.46–0.79)	0.75 (0.59–0.94)

RASi, renin-angiotensin system inhibitors; ACEi, angiotensin-converting enzyme inhibitors; ARB, angiotensin receptor blockers; ICI, immune checkpoint inhibitors; AHs, antihypertensives; LC, lung cancer; NSCLC, non-small cell lung cancer; SCLC, small cell lung cancer; RCT, randomized controlled trials; PD-1, programmed cell death protein 1; PD-L1, programmed cell death ligand 1; CTLA-4, cytotoxic t-lymphocyte-associated protein 4; HR, hazard ratio; OS, overall survival; PFS, progression-free survival; NR, not reported. For Kichenadasse et al., the HR shown in this table represents the summary estimate reported in the original publication. Individual trial-specific estimates (OAK, POPLAR, BIRCH, and FIR) were used in the meta-analysis because these represented independent randomized trial cohorts.

Most included investigations adopted retrospective cohort designs across different settings, including institutional cohorts, multicenter collaborations, and nationwide administrative datasets. One study represented a pooled *post hoc* analysis derived from randomized clinical trials. Study size varied substantially, ranging from 107 to 38,176 participants, with one nationwide French cohort contributing the majority of the available population.

Across studies, the proportion of patients receiving concomitant RAS inhibitors ranged from 13.3% to 46.9%. The study populations predominantly comprised advanced NSCLC cases, although one cohort specifically included SCLC patients and several studies included broader lung cancer populations without histological restriction.

Definitions of RAS inhibitor exposure were generally anchored to the period surrounding cancer diagnosis or initiation of ICI treatment, although operational criteria differed modestly across studies. Both ACE inhibitors and ARB were represented. Immunotherapy regimens mainly included PD-1 or PD-L1 inhibitors—most commonly nivolumab, pembrolizumab, atezolizumab, and durvalumab—administered either alone or in combination with systemic therapies. Treatment settings encompassed first-line and later-line use.

Geographically, the evidence base reflected broad international representation and included studies conducted in Asia (China and Japan), Europe (France and Portugal), North America (United States), and Turkey.

Overall methodological quality was acceptable to high, with scores ranging from 5 to 9 and twelve studies achieving scores ≥7. A detailed summary of the quality assessment is presented in **Supplementary Table 2**.

### Meta-analysis of survival outcomes and assessment of small-study effects

**Figure 2:**
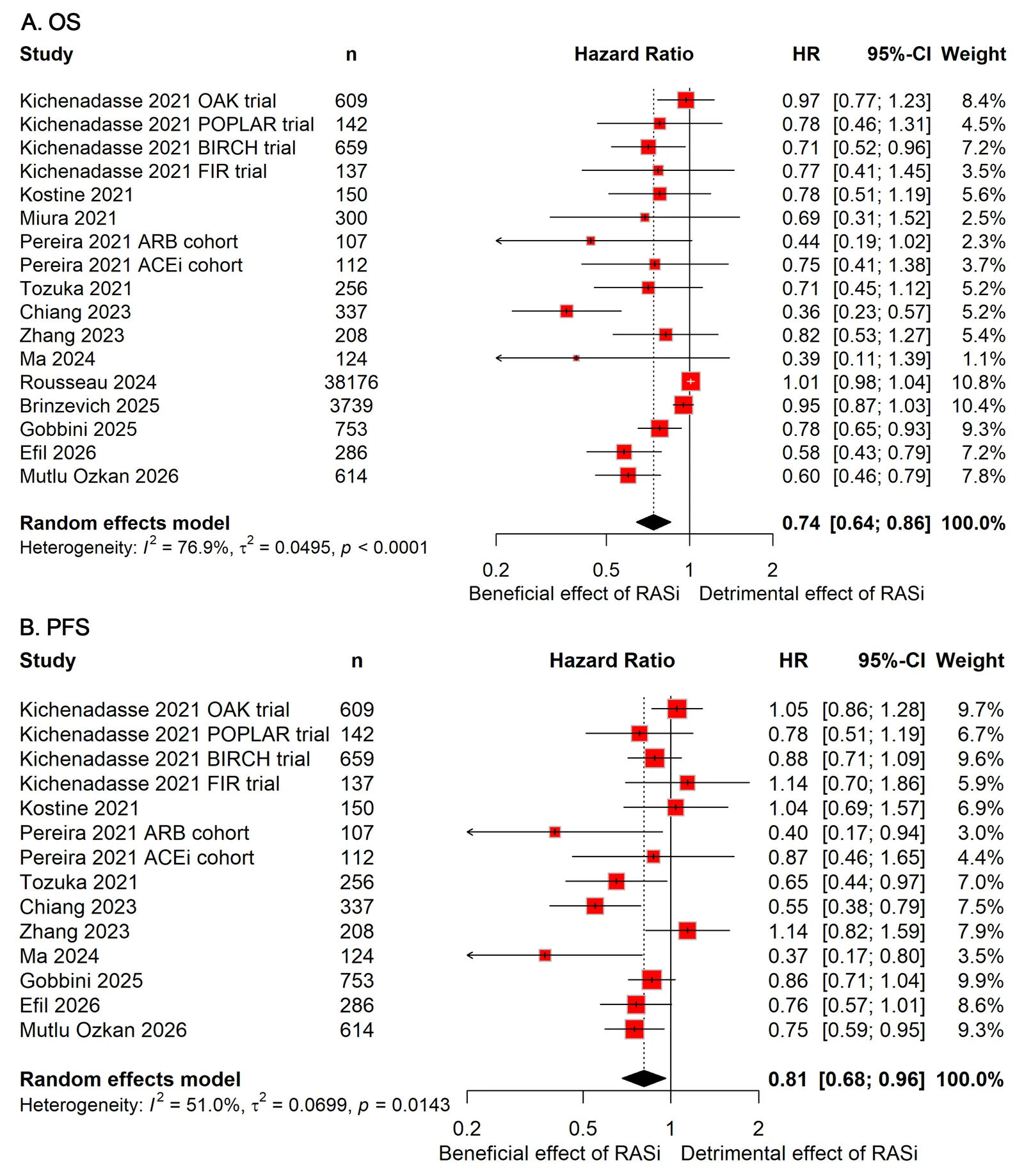
Forest plots of the conventional random-effects meta-analysis for **(A)** overall survival and **(B)** progression-free survival.

Using conventional random-effects pooling, concomitant exposure to RAS inhibitors was associated with improved survival outcomes among lung cancer patients treated with ICIs. The pooled HR for OS was 0.74 (95% CI 0.64–0.86; *I²* = 76.9%; **Figure 2A**), while the pooled HR for PFS was 0.81 (95% CI 0.68–0.96; *I²* = 51.0%; **Figure 2B**).

Because substantial heterogeneity and visual funnel asymmetry were observed, additional analyses were undertaken to evaluate the potential influence of small-study effects. Egger’s regression suggested asymmetry for OS (*p* < 0.0001). Therefore, PET-PEESE was performed as an exploratory sensitivity approach rather than a replacement for the primary pooled analysis.

**Figure 3:**
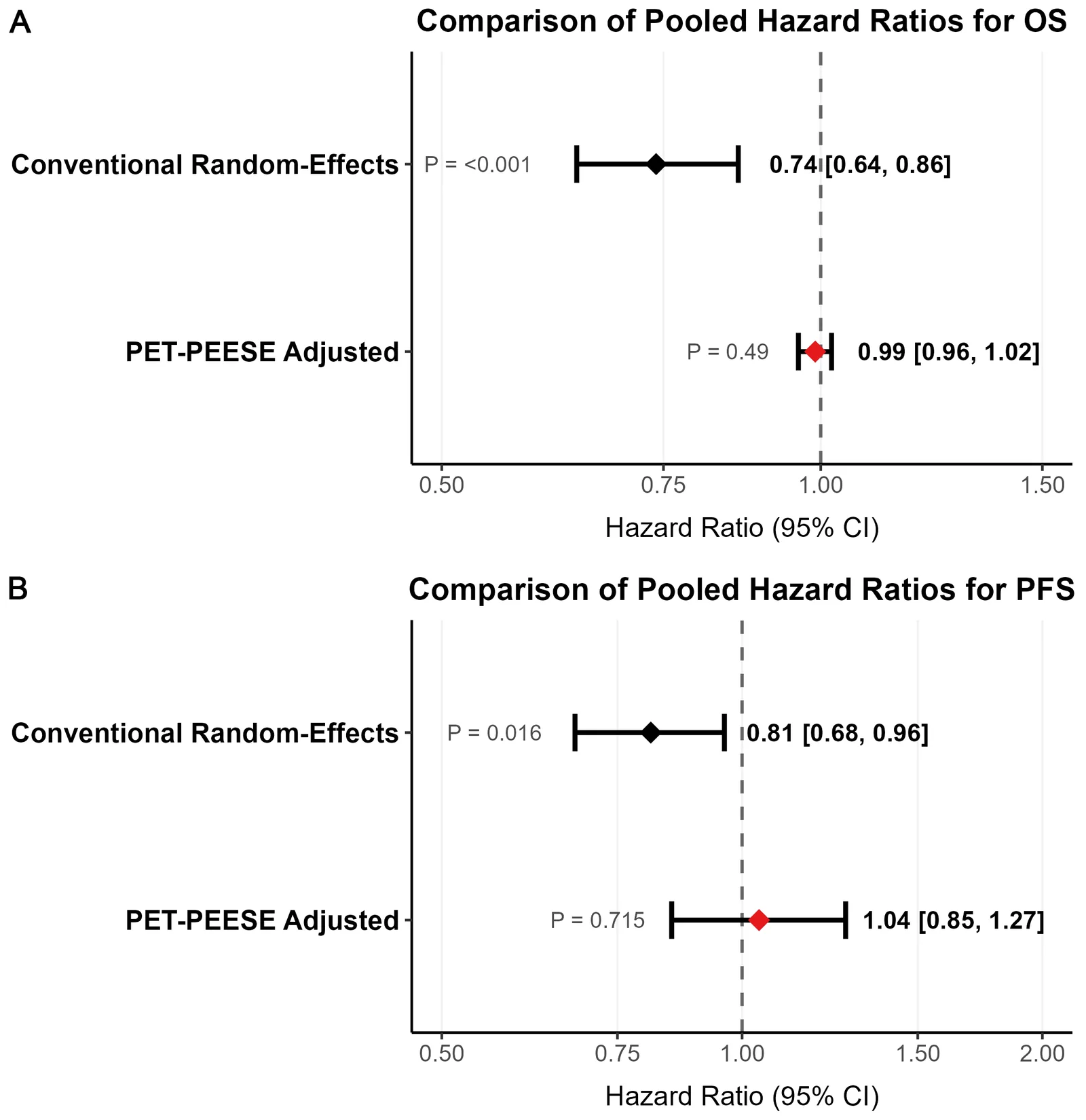
Comparison of pooled hazard ratios between conventional random-effects model and PET-PEESE adjusted model. **(A)** Overall survival (OS). **(B)** Progression-free survival (PFS).

**Figure 4:**
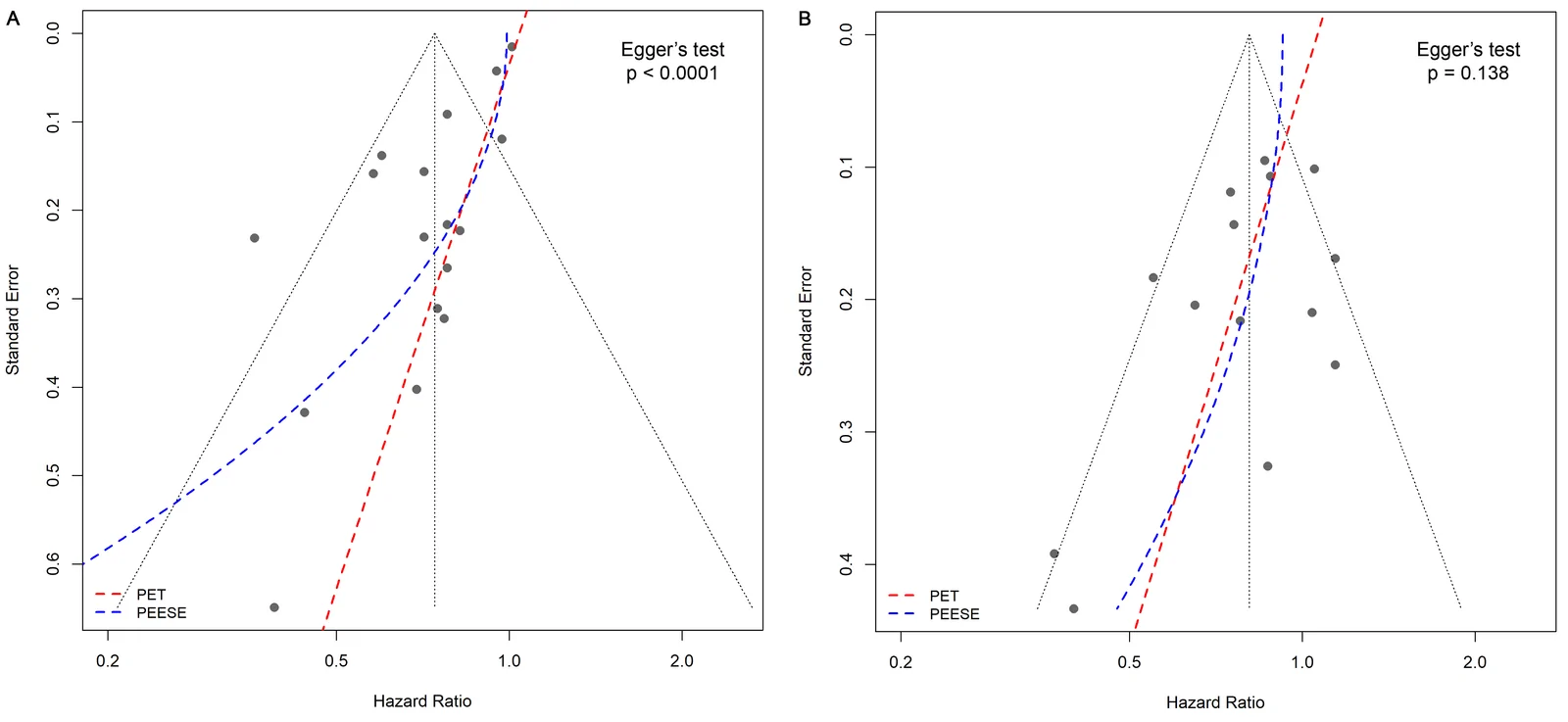
Funnel plots for **(A)** overall survival **(B)** progression-free survival with PET and PEESE regression lines.

After adjustment, the estimated association for OS moved toward the null (adjusted HR 0.99, 95% CI 0.96–1.02; **Figure 3A**). A similar pattern was observed for PFS (adjusted HR 1.04, 95% CI 0.85–1.27; **Figure 3B**). Funnel plots together with PET and PEESE regression models are shown in **Figure 4**.

**Figure 5:**
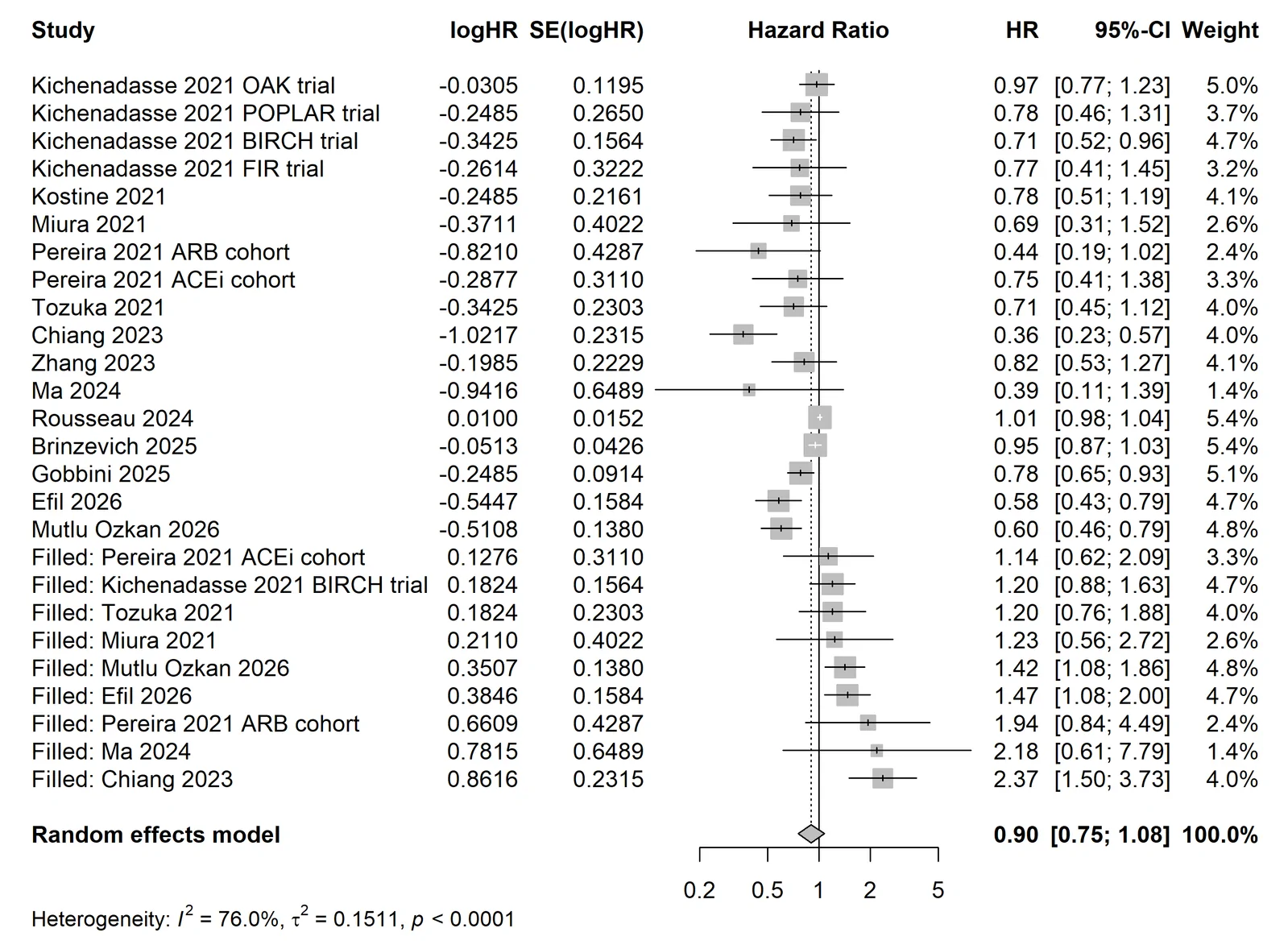
Trim-and-fill analysis assessing the potential influence of small-study effects on the association between RAS inhibitor exposure and overall survival in lung cancer patients receiving immune checkpoint inhibitors.

To further evaluate the robustness of the observed small-study effects, an additional trim-and-fill analysis was performed for OS. After applying the trim-and-fill adjustment, the pooled estimate was attenuated from HR 0.74 to HR 0.90 (95% CI 0.75–1.08), with the CI crossing the null value (**Figure 5**). The corresponding funnel plot with imputed studies is presented in **Supplementary Figure 2**.

Collectively, both PET-PEESE and trim-and-fill analyses reduced the magnitude of the association observed in the conventional random-effects model, suggesting that the apparent survival advantage associated with RAS inhibitor exposure may be partly influenced by small-study effects.

### Subgroup analysis according to tumor histology

Prespecified subgroup analyses were conducted to explore whether histological composition modified the observed associations.

**Figure 6:**
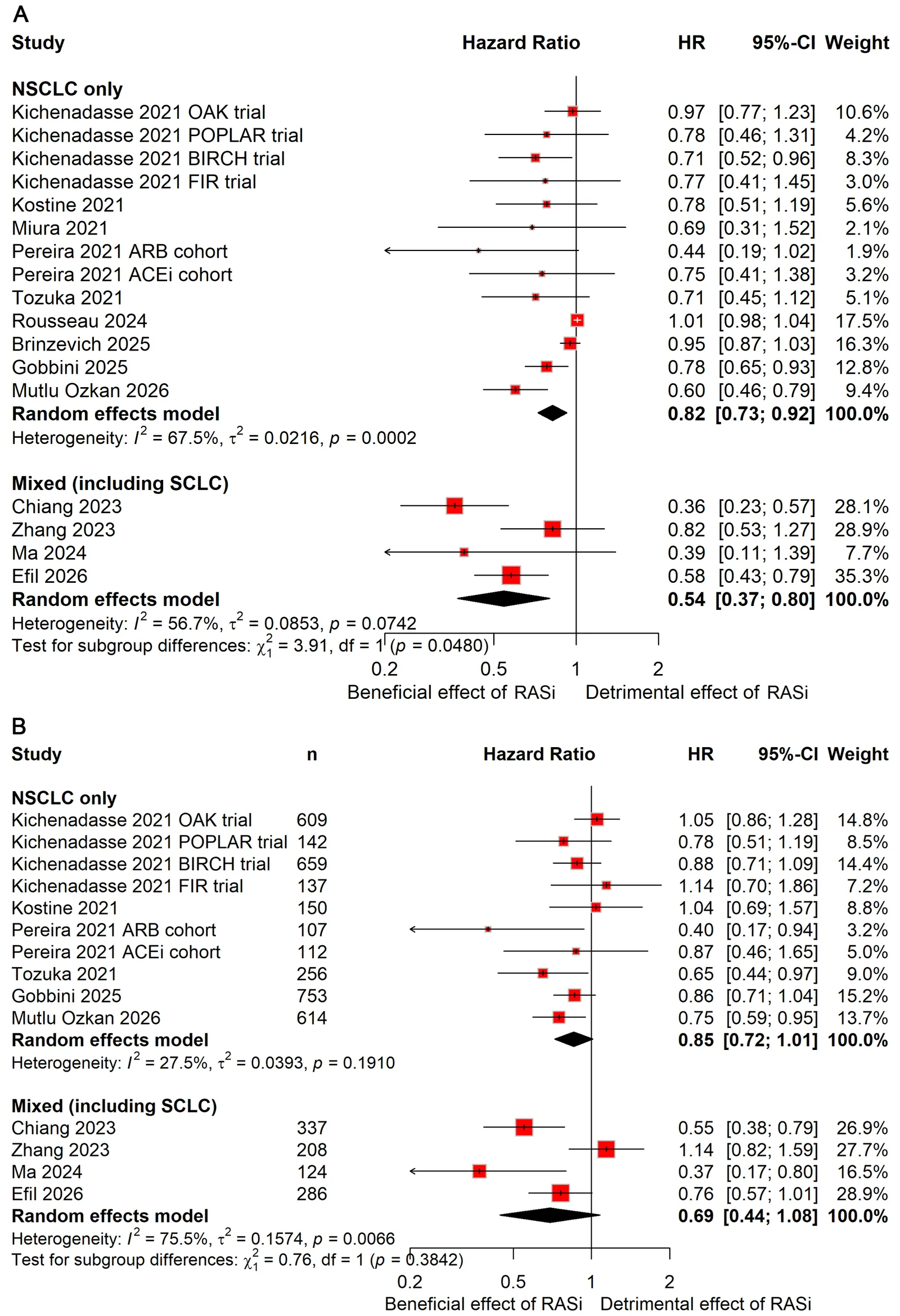
Forest plot of subgroup analysis for **(A)** overall survival **(B)** progression-free survival stratified by tumor histology.

For OS, studies restricted to NSCLC showed a pooled HR of 0.82 (95% CI 0.73–0.92), whereas studies including mixed histology populations yielded a pooled HR of 0.54 (95% CI 0.37–0.80). Evidence for between-subgroup heterogeneity reached nominal statistical significance (interaction *p* = 0.048; **Figure 6A**).

For PFS, pooled HRs were 0.85 (95% CI 0.72–1.01) and 0.69 (95% CI 0.44–1.08) in the NSCLC-only and mixed-histology groups, respectively, without statistically significant subgroup interaction (*p* = 0.384; **Figure 6B**).

Given the limited number of studies and imbalance in subgroup composition, these analyses should be considered exploratory and hypothesis-generating.

### Subgroup analysis according to geographic region

Regional subgroup analyses were further performed to assess potential geographic variation.

**Figure 7:**
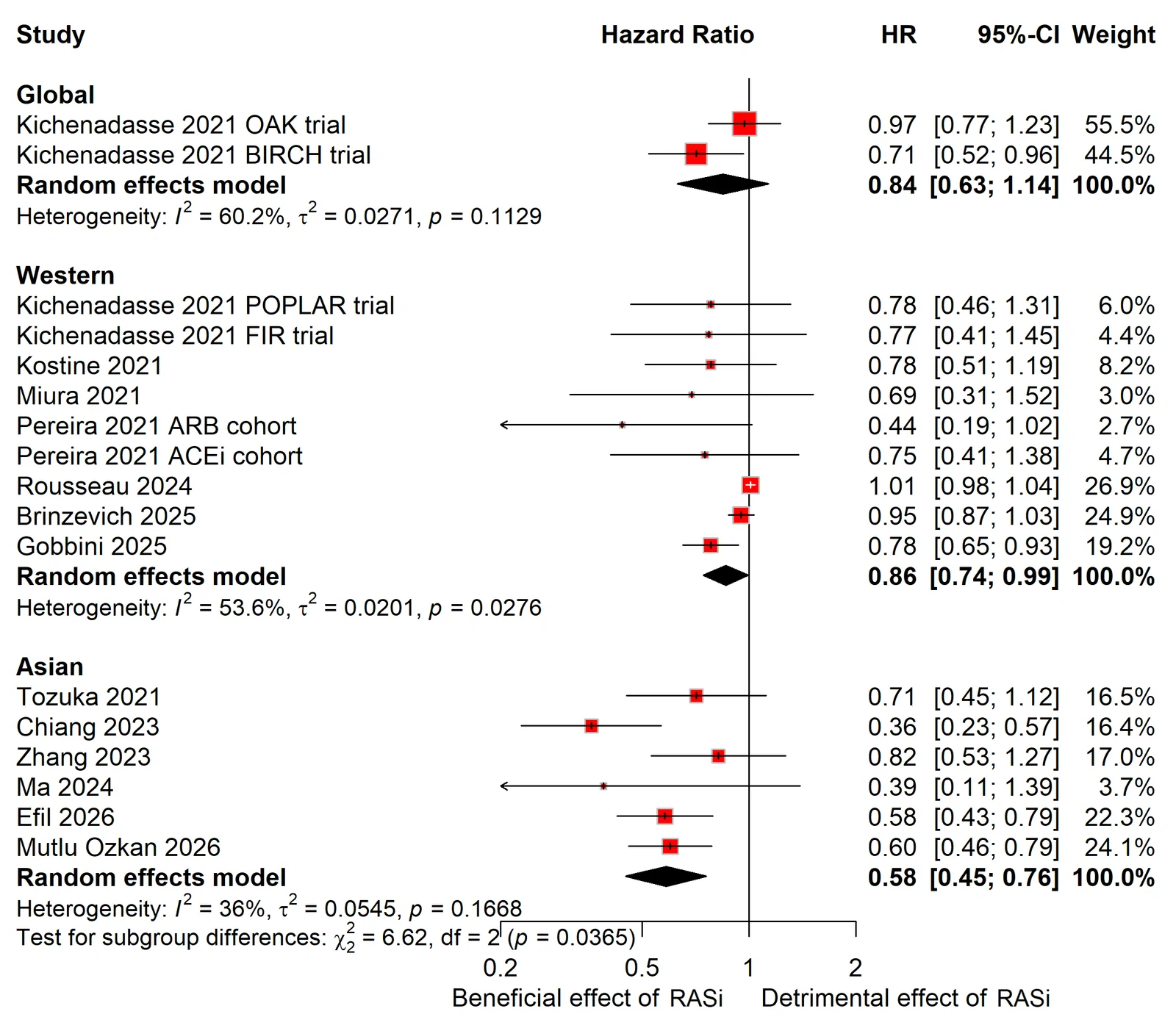
Forest plot of subgroup analysis for overall survival stratified by geographic region.

For OS, pooled HR estimates were 0.84 (95% CI 0.63–1.14) in globally recruited populations, 0.86 (95% CI 0.74–0.99) in Western cohorts, and 0.58 (95% CI 0.45–0.76) in Asian cohorts. Statistical evidence for subgroup heterogeneity was observed (interaction *p* = 0.036; **Figure 7**).

**Figure 8:**
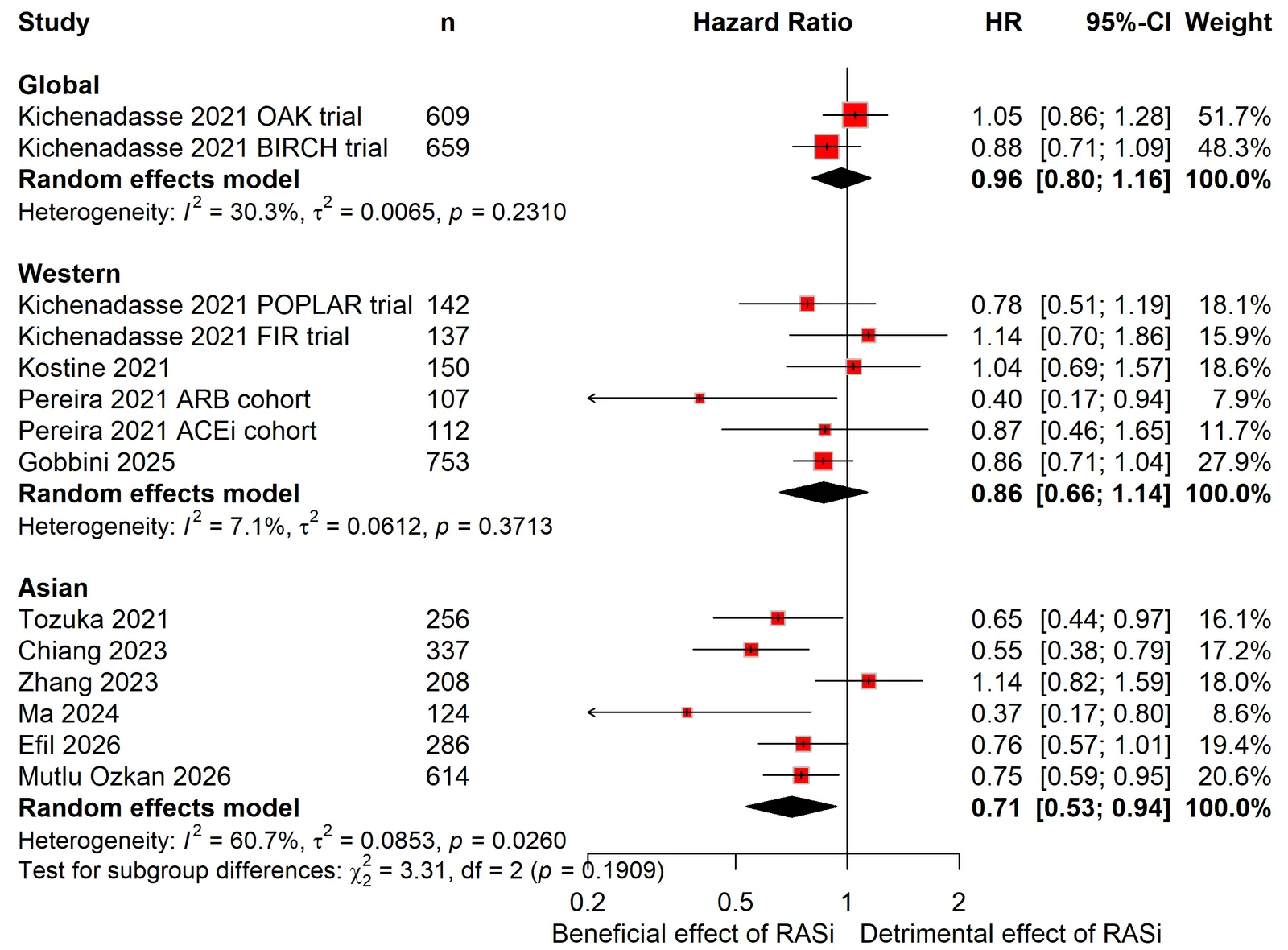
Forest plot of subgroup analysis for progression-free survival stratified by geographic region.

For PFS, pooled HRs were 0.96 (95% CI 0.80–1.16), 0.86 (95% CI 0.66–1.14), and 0.71 (95% CI 0.53–0.94), respectively, with no evidence of subgroup interaction (*p* = 0.191; **Figure 8**).

Because regional categories may capture multiple differences in patient selection, clinical practice, exposure definition, and residual confounding, these findings should not be interpreted as evidence of population-specific biological responsiveness.

### Sensitivity analysis

Leave-one-out analyses were conducted to evaluate the influence of individual studies on pooled estimates. Sequential exclusion of single studies resulted in only modest variation in pooled effect sizes. OS estimates ranged from 0.72 to 0.79, and PFS estimates ranged from 0.79 to 0.84 (**Supplementary Figure 1**). No individual study materially altered the overall direction of association. These findings indicate that the conventional pooled estimates were not dominated by any single dataset. However, stability under leave-one-out procedures does not exclude systematic sources of bias that may operate across multiple studies simultaneously. Therefore, the attenuation observed in PET-PEESE analyses should be interpreted as complementary information regarding potential small-study effects rather than inconsistency of the sensitivity analyses.

### Complementary transcriptomic analyses of *AGTR1*-associated CAF biology

**Figure 9:**
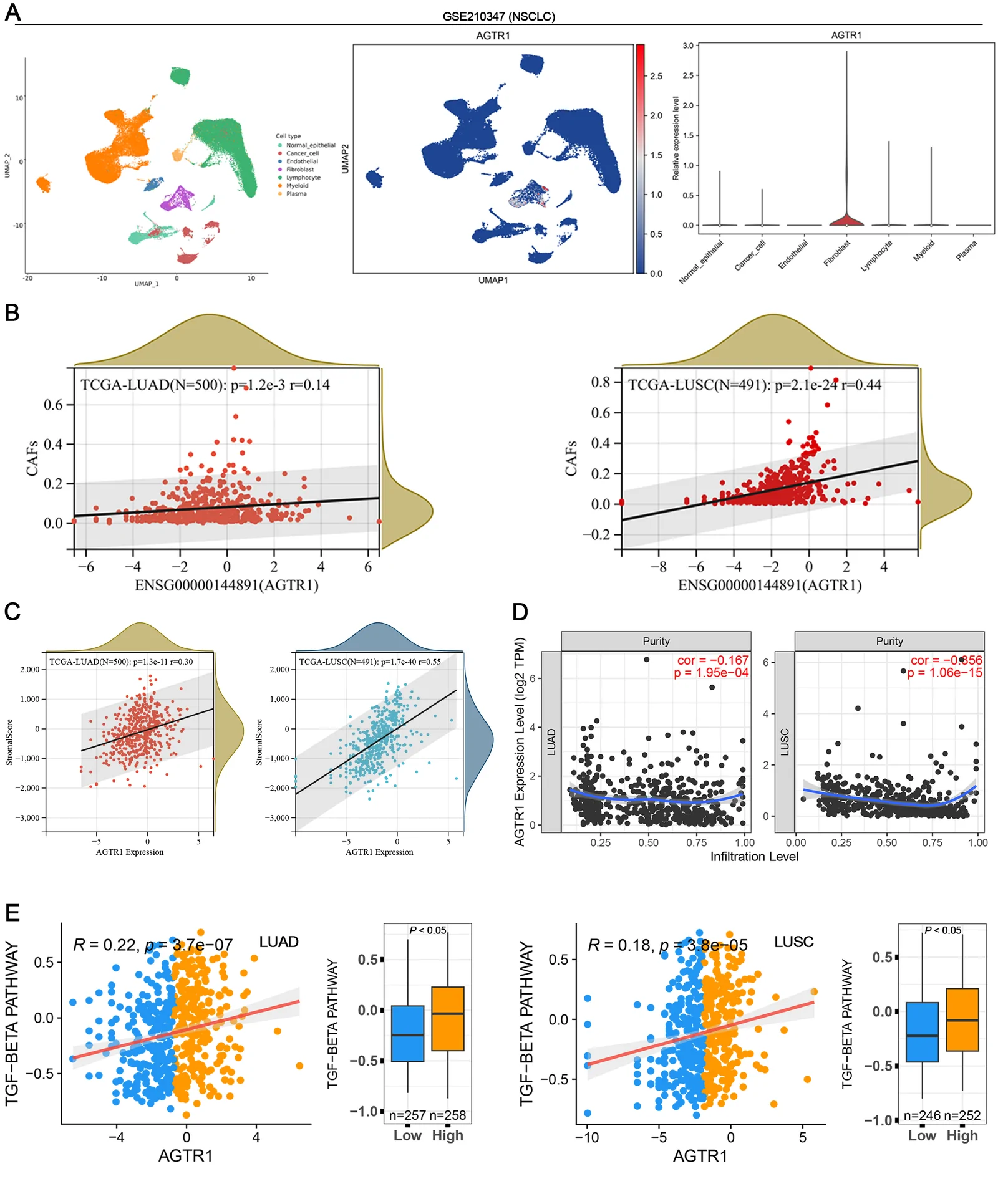
Complementary transcriptomic analyses characterizing *AGTR1*-associated CAF biology in lung cancer. **(A)** scRNA-seq shows specific high *AGTR1* expression in NSCLC fibroblasts. **(B)** *AGTR1* expression positively correlates with CAF abundance. **(C)** *AGTR1* expression positively correlates with stromal score. **(D)** *AGTR1* expression correlates with tumor purity. **(E)** *AGTR1* expression positively correlates with TGF-β pathway activity.

Analysis of the GSE210347 scRNA-seq dataset demonstrated that *AGTR1* expression was enriched in fibroblast populations within the TME (**Figure 9A**). Consistent with this finding, *AGTR1* expression was positively correlated with EPIC-estimated CAF infiltration in the TCGA LUAD and LUSC cohorts (**Figure 9B**), supporting an association between *AGTR1* expression and CAF-enriched tumor stroma.

To further characterize the stromal context of *AGTR1* expression, correlations with stromal abundance and tumor purity were evaluated. *AGTR1* expression showed a significant positive correlation with StromalScore (**Figure 9C**) and a corresponding negative correlation with tumor purity (**Figure 9D**), indicating that elevated *AGTR1* expression is associated with a stromal-enriched TME.

Given the established role of TGF-β signaling in CAF activation and extracellular matrix remodeling, we further examined the association between *AGTR1* expression and TGF-β pathway activity. *AGTR1* expression was positively correlated with TGF-β signaling activity in both LUAD and LUSC cohorts (**Figure 9E**), consistent with the notion that *AGTR1* expression is associated with stromal activation and CAF-related TME characteristics.

**Figure 10:**
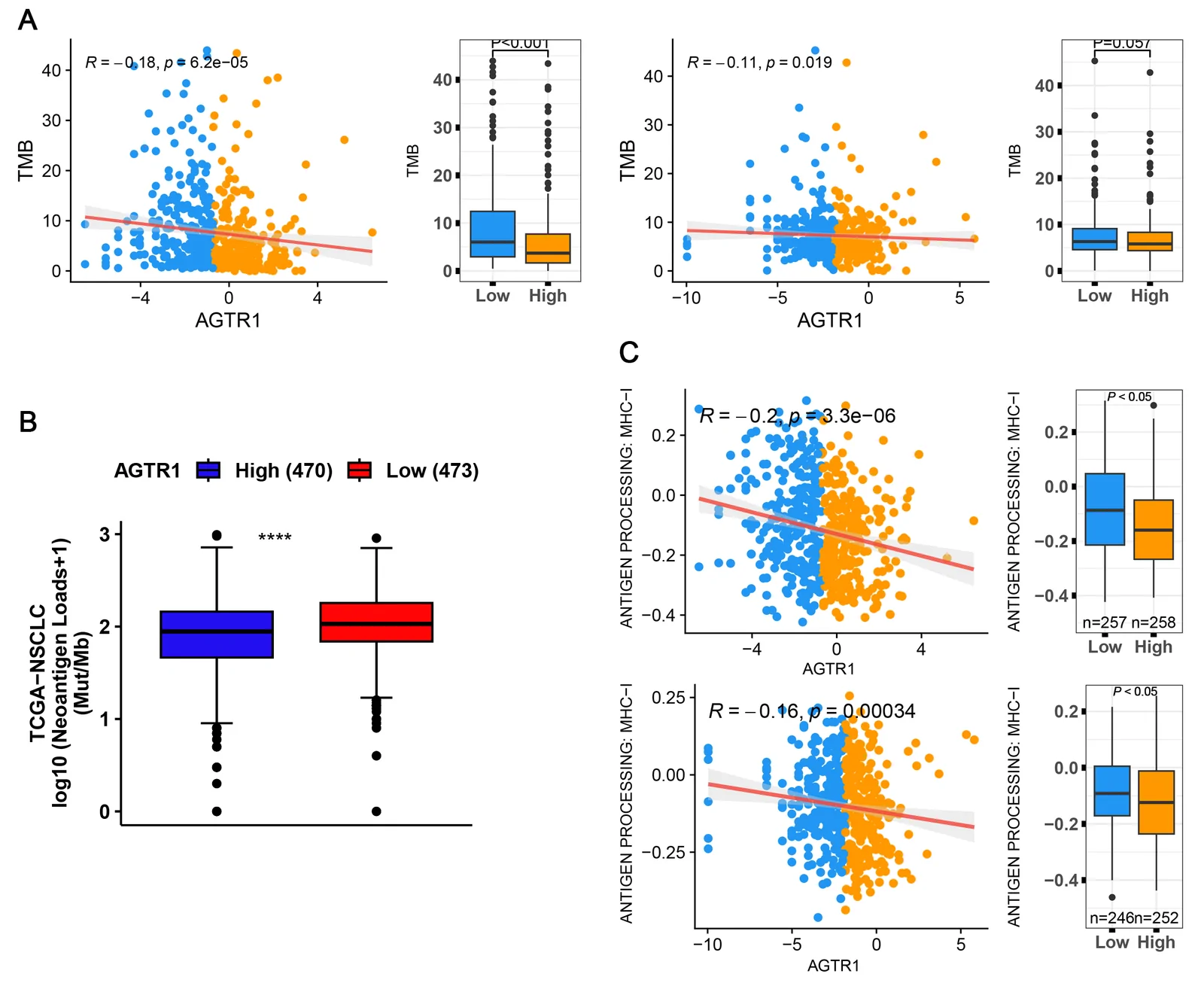
Association between *AGTR1* expression and tumor immunogenicity-related features in lung cancer. **(A)** *AGTR1* expression negatively correlates with tumor mutational burden (TMB). **(B)** Low *AGTR1* expression is associated with higher neoantigen loads. **(C)** *AGTR1* expression negatively correlates with antigen processing MHC-I scores.

*AGTR1* expression was additionally inversely correlated with tumor immunogenicity-related features, including tumor mutation burden, neoantigen load, and MHC-I pathway activity (**Figure 10**).

Collectively, these complementary transcriptomic analyses provide independent biological support for the proposed RAS–CAF framework, suggesting that *AGTR1* expression marks a stromal-enriched and less immunogenic TME characterized by CAF accumulation, TGF-β pathway activation, and reduced antigen presentation features. However, these analyses remain associative and do not establish a causal relationship between *AGTR1* signaling, CAF biology, and the clinical outcomes observed in the present meta-analysis.

## Discussion

To our knowledge, this is the first lung cancer-specific systematic review and meta-analysis examining the association between concomitant RAS inhibitor exposure and outcomes among patients receiving ICIs, and currently represents the largest evidence synthesis addressing this clinically relevant question, incorporating data from 46,618 patients across 13 studies. Although conventional random-effects pooling suggested significant improvements in both OS and PFS, exploratory assessment of small-study effects substantially attenuated these associations. Collectively, these findings indicate that the apparent survival advantage observed in conventional meta-analysis should be interpreted cautiously and may not represent a reproducible treatment effect across unselected patient populations.

The present findings should also be interpreted in the context of the growing interest in CAF-mediated remodeling of the TME. Experimental evidence has implicated RAS signaling in stromal activation, extracellular matrix remodeling, and immune regulation, providing a biological rationale for investigating whether RAS inhibition could modify responsiveness to ICIs (11, 13, 15, 42, 43). Because CAFs exhibit substantial functional heterogeneity, however, any immunomodulatory consequences of RAS inhibition are likely to be context dependent rather than universally applicable. In line with this concept, our complementary transcriptomic analyses indicated that *AGTR1* expression was preferentially associated with fibroblast-enriched and stromal-active tumor states, accompanied by enhanced TGF-β pathway activity and reduced immunogenic features. These findings provide a potential biological framework linking RAS-related signaling with CAF-associated immune regulation, while highlighting the complexity of predicting clinical benefit from RAS inhibition based solely on drug exposure.

Although the complementary transcriptomic analyses support the biological plausibility of a RAS–CAF axis, the present clinical meta-analysis cannot establish whether the observed survival associations are mechanistically attributable to CAF biology. None of the included studies incorporated tumor-level measurements of CAF phenotypes, stromal architecture, immune infiltration, or pathway activity. Therefore, although CAF-mediated mechanisms remain biologically plausible, they should currently be regarded as hypotheses rather than conclusions supported by the available clinical evidence.

Instead, our results suggest that if interactions between RAS signaling and immunotherapy response exist, they are unlikely to operate uniformly across broad clinical populations and may depend on underlying tumor context.

Exploratory subgroup analyses identified nominal differences according to histological composition and geographic region. In particular, larger effect estimates appeared in studies including mixed histology cohorts and in Asian populations. However, these observations should be interpreted conservatively. Subgroup analyses were underpowered, not adjusted for multiplicity, and vulnerable to ecological confounding. Factors such as treatment sequencing, healthcare systems, comorbidity patterns, and exposure definitions may contribute to apparent subgroup variation. Because subgroup allocation largely paralleled differences in study size, these exploratory findings should not be interpreted as evidence of biological effect modification. These observations therefore warrant independent validation rather than immediate biological interpretation.

Several limitations should also be acknowledged. First, all included evidence originated predominantly from observational datasets and therefore remains susceptible to residual confounding and confounding by indication. Second, exposure definitions differed across studies with respect to the timing and definition of RAS inhibitor ascertainment, and detailed information regarding treatment duration, dose intensity, and longitudinal exposure was generally unavailable. Although most studies defined exposure at or around cancer diagnosis or initiation of ICI therapy, one study ascertained RAS inhibitor use after initiation of immunotherapy, introducing the potential for immortal time bias. To evaluate the influence of this methodological difference, we performed an additional sensitivity analysis excluding this study, and the pooled estimate remained materially unchanged (HR 0.79, 95% CI 0.70–0.89), although between-study heterogeneity decreased modestly (I² from 76.9% to 70.4%). Nevertheless, differences in exposure ascertainment may still have contributed to between-study heterogeneity and should be considered when interpreting the pooled findings. Third, patient-level biomarker data were lacking, precluding evaluation of potential interactions with PD-L1 expression, molecular subtype, or stromal phenotypes. Fourth, baseline demographic characteristics and comorbidities were not consistently reported across the included studies. In particular, information regarding age, sex, hypertension, cardiovascular disease, diabetes mellitus, and chronic kidney disease was unavailable or incompletely adjusted for in several studies. Because these factors may influence both the likelihood of receiving RAS inhibitors and clinical outcomes, their contribution to between-study heterogeneity and residual confounding could not be fully evaluated. Fifth, one included study reported separate adjusted estimates for ACE inhibitor and ARB exposure derived from the same underlying cohort. These exposure-specific estimates were analyzed separately to preserve the original exposure definitions reported by the investigators; however, because they shared a common reference population, complete statistical independence cannot be assumed. Although this study contributed only a small proportion of the overall sample size, separate inclusion of these estimates may have resulted in a modest underestimation of variance and should therefore be considered when interpreting the pooled results. In addition, for several studies evaluating ACE inhibitors or ARBs separately, the composition of the comparator group was not fully described, making it impossible to determine whether exposure to the alternative RAS inhibitor class was present. Although such exposure contamination could not be confirmed from the published reports, potential exposure misclassification cannot be excluded and may have attenuated the observed associations. Additionally, RAS inhibitors represent only one of several classes of concomitant medications that have been investigated for their potential influence on ICI efficacy. Furthermore, in several included studies, RAS inhibitor exposure was evaluated as one of multiple concomitant medications rather than as the primary research question. This secondary-exposure design may increase the potential for selective reporting of medication-outcome associations and may partially contribute to the observed small-study effects. Other commonly prescribed agents, including antibiotics, corticosteroids, proton pump inhibitors, metformin, statins, and acetaminophen, have likewise been associated with variable immunotherapy outcomes (44–47). Because the included studies generally did not comprehensively account for the use of these concomitant medications, the independent contribution of RAS inhibition to the observed clinical outcomes cannot be fully distinguished. Finally, although PET-PEESE and trim-and-fill provide useful exploratory approaches for assessing small-study effects, their performance under conditions of substantial between-study heterogeneity remains debated.

From a clinical perspective, these findings do not support the use of RAS inhibitors as a strategy to enhance immunotherapy efficacy in unselected lung cancer populations. At the same time, continued RAS inhibitor exposure for established cardiovascular indications does not appear to compromise ICI outcomes based on currently available evidence. Decisions regarding continuation of antihypertensive therapy should therefore continue to be guided primarily by established cardiovascular indications rather than expectations of anticancer synergy.

Future investigations should move beyond conventional exposure–outcome associations toward biomarker-informed translational studies integrating clinical outcomes with characterization of the TME. Prospective studies incorporating spatial transcriptomics, single-cell profiling, and longitudinal tissue sampling may help determine whether specific CAF phenotypes or stromal states identify patient subsets that could potentially benefit from RAS modulation during immunotherapy.

## Conclusion

Current evidence does not support a consistent survival advantage associated with concomitant RAS inhibitor use during ICI therapy in unselected patients with lung cancer. Although conventional meta-analysis suggested favorable associations, exploratory analyses accounting for small-study effects substantially attenuated these findings, indicating that the observed survival advantage may partly reflect systematic bias and residual confounding. Future prospective studies integrating clinical outcomes with comprehensive TME profiling—including characterization of CAF phenotypes, stromal architecture, and immune cell composition—are warranted to determine whether biologically defined patient subsets, rather than unselected populations, may derive benefit from RAS inhibition in combination with immunotherapy.

## Data Availability

The original contributions presented in the study are included in the article/**Supplementary Material**. Further inquiries can be directed to the corresponding author.
