# Supplementary material for: Renin–angiotensin system inhibitors and immunotherapy outcomes in lung cancer: a systematic review and meta-analysis with complementary transcriptomic analyses
Source: Front Immunol. 2026 Aug 19;17:1925741. doi: 10.3389/fimmu.2026.1925741 (PMC13534140; doi:10.3389/fimmu.2026.1925741)
Supplement: Supplementary file 1 [file DataSheet1.docx]

**Supplementary Material**

**Renin–angiotensin system inhibitors and immunotherapy outcomes in lung cancer: a systematic review and meta-analysis with complementary transcriptomic analyses**

**Supplementary Table 1.** Details of search strategy.

**Supplementary Table 2.** Quality assessment of included studies through the Newcastle-Ottawa Scale.

**Supplementary Figure 1.** Leave-one-out sensitivity analysis for overall survival and progression-free survival.

**Supplementary Figure 2.** Funnel plot with trim-and-fill imputation assessing potential small-study effects for overall survival.

**Supplementary Table 1.** Details of search strategy.

| **Database** | **Search strategy** |
| --- | --- |
| ***PubMed*** | ("Renin-Angiotensin System"[Mesh] OR "Angiotensin-Converting Enzyme Inhibitors"[Mesh] OR "Angiotensin Receptor Antagonists"[Mesh] OR "Angiotensin-Converting Enzyme Inhibitors" OR "Angiotensin Receptor Antagonists" OR ACEI OR ACEIs OR ARB OR ARBs OR "ACE inhibitor*" OR "angiotensin converting enzyme inhibitor*" OR "angiotensin receptor blocker*" OR "angiotensin receptor antagonist*" OR "RAS inhibitor*" OR "RAAS inhibitor*" OR "renin angiotensin system inhibitor*" OR "renin angiotensin aldosterone system inhibitor*" OR RAAS OR RAASi OR enalapril OR lisinopril OR ramipril OR captopril OR benazepril OR fosinopril OR quinapril OR moexipril OR perindopril OR trandolapril OR losartan OR valsartan OR irbesartan OR candesartan OR olmesartan OR telmisartan OR eprosartan OR azilsartan OR sacubitril OR "sacubitril valsartan") AND ("Immune Checkpoint Inhibitors"[Mesh] OR "immune checkpoint inhibitor*" OR "immune checkpoint blockade" OR ICI OR ICIs OR ICB OR "PD-1" OR "PD-L1" OR "CTLA-4" OR "PD-1 inhibitor*" OR "PD-L1 inhibitor*" OR "CTLA-4 inhibitor*" OR nivolumab OR pembrolizumab OR atezolizumab OR durvalumab OR avelumab OR cemiplimab OR ipilimumab OR tremelimumab OR dostarlimab OR tislelizumab OR sintilimab OR camrelizumab OR toripalimab) AND ("Neoplasms"[Mesh] OR "Carcinoma, Non-Small-Cell Lung"[Mesh] OR "lung cancer" OR "non-small cell lung cancer" OR NSCLC OR "small cell lung cancer" OR SCLC OR cancer)  **Search items: 128** |
| ***Embase*** | ('renin angiotensin system'/exp OR 'angiotensin converting enzyme inhibitor'/exp OR 'angiotensin ii receptor antagonist'/exp OR acei OR arb OR raas OR raasi OR 'ace inhibitor*' OR 'angiotensin receptor blocker*' OR enalapril OR lisinopril OR ramipril OR captopril OR losartan OR valsartan OR irbesartan OR candesartan OR telmisartan) AND ('immune checkpoint inhibitor'/exp OR 'pd-1'/exp OR 'pd-l1' OR 'ctla-4'/exp OR nivolumab OR pembrolizumab OR atezolizumab OR durvalumab OR ipilimumab OR tislelizumab OR sintilimab OR camrelizumab OR toripalimab) AND (('lung cancer'/exp OR 'non small cell lung cancer'/exp OR lung) AND cancer OR nsclc)  **Search items: 402** |

**Supplementary Table 2.** Quality assessment of included studies through the Newcastle-Ottawa Scale.

| **Study** | **Selection (0–4)** | **Comparability (0–2)** | **Outcome (0–3)** | **Total (0–9)** | **Quality** |
| --- | --- | --- | --- | --- | --- |
| Kichenadasse et al. 2021 | 4 | 2 | 3 | 9 | High |
| Kostine et al. 2021 | 3 | 1 | 3 | 7 | High |
| Miura et al. 2021 | 3 | 2 | 2 | 7 | High |
| Pereira et al. 2021 | 2 | 0 | 3 | 5 | Moderate |
| Tozuka et al. 2021 | 3 | 2 | 2 | 7 | High |
| Chiang et al. 2023 | 3 | 2 | 3 | 8 | High |
| Zhang et al. 2023 | 3 | 1 | 3 | 7 | High |
| Ma et al. 2024 | 3 | 2 | 3 | 8 | High |
| Rousseau A, et al. 2024 | 4 | 2 | 3 | 9 | High |
| Brinzevich et al. 2025 | 4 | 2 | 3 | 9 | High |
| Gobbini et al. 2025 | 3 | 2 | 3 | 8 | High |
| Efil et al. 2026 | 3 | 2 | 3 | 8 | High |
| Mutlu Ozkan et al. 2026 | 3 | 2 | 3 | 8 | High |

Notes: Selection: representativeness of exposed cohort, selection of non‑exposed cohort, ascertainment of exposure, demonstration that outcome was not present at start. Comparability: control for important confounders (e.g., age, sex, performance status, comorbidities, treatment line). Outcome: assessment of outcome, adequacy of follow‑up duration, completeness of follow‑up.

**Supplementary Figure 1.** Leave-one-out sensitivity analysis for (**A**) overall survival and (**B**) progression-free survival.

**Supplementary Figure 2.** Funnel plot with trim-and-fill imputation assessing potential small-study effects for overall survival.
